# Climate Change and Potential Health Effects in Mexican Children

**DOI:** 10.29024/aogh.915

**Published:** 2018-07-27

**Authors:** Horacio Riojas-Rodríguez, María Laura Quezada-Jiménez, Pamela Zúñiga-Bello, Magali Hurtado-Díaz

**Affiliations:** 1 Environmental Health Department, Center for Population Health Research, National Institute of Public Health, Universidad No. 655 Colonia Santa María Ahuacatitlán, C.P. 62100, Cuernavaca, Morelos, MX; 2 Academic Secretary, National Institute of Public Health, Universidad No. 655 Colonia Santa María Ahuacatitlán, C.P. 62100, Cuernavaca, Morelos, MX; 3 Academic Secretary, School of Tourism, Autonomous University of Morelos State (UAEM), Av. Universidad 1001, Col. Chamilpa C.P. 62210, Cuernavaca, Morelos, MX

## Abstract

Climate change (CC) is the most important challenge of our time, a long-term global problem and one of the most serious global threats to human health in the future. CC is the expression of changes in temperature and water cycle, floods and drought events, extreme heat waves and sea level rise. Children are particularly vulnerable because they are highly sensitive to climate changes. The main environmental hazards affecting children’s health are poor household drinking water quality and availability, lack access to adequate sanitary facilities, poor hygiene practices, outdoor and indoor air pollution, vector-borne diseases, chemical hazards, and unintentional injuries. Except for some unintentional injuries, these environmental hazards are associated to CC.

## Introduction

Climate change (CC) is the most important challenge of our time, a long-term global problem and one of the most serious global threats to human health in the future [1]. CC is the expression of changes in temperature and water cycle, floods and drought events, extreme heat waves and sea level rise [2]. Children are particularly vulnerable because they are highly sensitive to climate changes. The main environmental hazards affecting children’s health are poor household drinking water quality and availability, lack of access to adequate sanitary facilities, poor hygiene practices, outdoor and indoor air pollution, vector-borne diseases, chemical hazards, and unintentional injuries. Except for some unintentional injuries, these environmental hazards are associated with CC [3].

## The Burden of Disease Attributable to Climate Change in Children

CC is one of the main environmental risks of this century, contributing to the increase of the burden of disease, particularly in poor and developing regions [3]. According to the World Health Organization (WHO), CC will cause approximately 250,000 additional deaths each year due to malnutrition, malaria and diarrhea during 2030–2050. From gestation throughout adolescence, children are vulnerable to environmental exposures during the critical windows of development. The United Nations International Children’s Emergency Fund (UNICEF) highlighted in 2014 that 80% of the deaths attributable to CC occur in children. Moreover, it is estimated that 500 million children and adolescents live in areas of high flood risk due to CC [4].

Globally, CC is expected to escalate to the top five causes of death in children under 5 years old [5]. Short-lived climate pollutants are particularly important for being climate forcers and having negative health effects, especially in vulnerable populations. For instance, increased ozone levels are associated with asthmatic episodes [6]. Additionally, CC will cause 4,000 malnutrition deaths in children less than 15 years old between 2030 and 2050, mainly in regions that depend on agriculture and livestock, and the number of malnourished children is expected to increase by 20% by 2050 [5]. Other impacts of CC on children’s health are acute diarrheal diseases [7], vector borne diseases [8] and the symptoms of post-traumatic stress disorder from extreme weather events [9].

In 2015 the world’s child population represented almost one third (625 millions) of the world’s population and nearly 2.2 billion were under five years old [4]. This is the future adult population, and their vulnerability to global warming compromises their development and the development of future generations.

## Climate Change in Mexico

Mexico is vulnerable to the effects of CC on human health [10]. It has been documented that temperature will increase by 4°C in the border with the United States and 2.5° to 3.5°C in the rest of the country, which will increase the probability of winter rains in the northeastern deserts of the country, reduce the force of summer monsoons in southern Mexico, and would seem to have an important effect on the probability of extreme hurricanes [24]. Natural disasters caused by rains, floods and tropical cyclones affected more than 7.8 million persons during the period of 2010–2013 [4]. Particularly during 2017, Hurricanes Irma, Harvey and Maria devastated Mexican territory in the Gulf of Mexico. According to UNICEF, Irma hurricane alone affected more than 10.5 million children and adolescents, including more than 3 million children under five years old [11].

CC has caused droughts affecting not only the northern and northeastern part of the country, but also the central region. It is estimated that 70% of Mexico’s territory is vulnerable to droughts, including Mexico City Metropolitan Area, Northern Sinaloa, the Basin of Conchos River and the Bajio region. Mexico’s farming surface is anticipated to suffer a reduction from 40% to 25%, which will impact its capacity to feed the population, placing children’s health in particular risk [12,13].

However, vulnerability arises not only from the geographic location or climate environments; environmental and socio-economic conditions are important factors triggering climate-sensitive illnesses, especially for vulnerable populations such as children.

## Potential Effects of Climate Change in the Health of Mexican Children

Childhood development is divided into three stages: early childhood (0–5 years old), school age (6–11 years old) and adolescence (12–17 years old), representing 32.4%, 33.7%, and 33.9% of the children population respectively [14].

In 2015, Mexico’s population under 18 years was 39.2 million, 51% boys and 49% girls. They represented 32.8% of the total population. However, these figures change within each state; in Chiapas and Guerrero – the poorest states in the country–children represent 39.4% and 37% of the population, respectively. Furthermore, 60% of the population under 18 years old lived in poverty, with 5.1 million children in extreme poverty [14]. In addition, 26.4% of the child population lived in rural areas, with their level of human development 9.9% below that of the children living in urban areas, and lacked access to education (one out of 10 children had not gone to school [4,15]. In addition, children and adolescents living in some northern and southeastern states of the country, such as Zacatecas, Nayarit, Chiapas, Guerrero, and Oaxaca – as well as indigenous communities – show still lower human development levels [4,15,16]. These characteristics magnify the potential impact of CC on children’s health.

By 2016, the general mortality rate for children under five years of age was 15.5%, and 12.7% for those less than one year old. However, in that same year, the main causes of mortality attributable to environmental causes in children less than five years old were diarrhea and lower respiratory tract infections, with a mortality rate of 14.78 deaths per 100,000. These diseases are also the main cause of mortality in the age group of 5–14 years old [17].

In Mexico, intestinal infections are the main causes of diarrhea in children and the second cause of morbidity (incidence rate of 41 cases per 100,000 inhabitants) [18]. In children less than a year old, 16,519 new cases per 100,000 were documented during 2013 [15]. A study in the Gulf of Mexico found a 22% increase in the weekly cases of ADD (CI 95%: 1.013–1.242) in children under five years old for every 1°C increase in maximum temperatures [19]. Meanwhile, the mortality rate for ADD was 28 deaths per 100,000 children under one year old [15,20]. Although these diseases have been associated with climate conditions, poverty and lack of hygiene are important conditioning factors [18].

Another effect in children associated with changes in temperature are acute respiratory tract infections (ARIs) [15]. They are the main cause of morbidity in Mexican children, with a rate of 230 cases per 100,000 inhabitants. The study carried out in the Gulf of Mexico mentioned above estimated that ARIs would diminish as temperatures rise because they appear to decrease with a 2- to 3-week lag following high temperatures [19].

Regarding nutritional issues, approximately 25% (11.7 million) of the Mexican children and adolescents live in food poverty [21]. According to the 2012 National Survey on Health and Nutrition (ENSANUT) 1,194,805 children suffer chronic malnutrition in the country. Chronic malnutrition in urban zones is 10.1%, while it rises to 19.9% in rural areas that depend on farming for survival. When crop food availability is reduced in these communities, they face an immediate risk. Additionally, the prevalence of chronic malnutrition is almost doubled in indigenous children [20]. Mortality caused by malnutrition in children under five years old is higher in the states that have a high social gap index (Oaxaca, Chiapas and Guerrero), along with Chihuahua, which in spite of having a low social Index, has 3% of its population living in the Tarahumara Mountains, where lack of access to adequate nutrition has serious effects: 50% of children under five years old suffer malnutrition, 23% have low weight and 2.5% are severely malnourished [22].

Dengue is the most documented vector-borne disease associated to CC in Mexico [23,24]. Its incidence increased from 5,220 cases in 2003 to 40, 559 in 2007, and 20.3% of this cases occurred in children from 5 to 14 years old [25]. In a study to evaluate the impact of climatic factors on the incidence of dengue in the municipalities of Veracruz, Mexico, the authors found that each 1°C increase in minimum temperature increased the dengue cases caused between 4.4% (CI 95%: (0.3%–8.4%) and 5.8% (CI 95%: 1.8%–9.8%) [24]. Other diseases such as Zika and Chikunguya could have similar behavior.

When addressing heat stroke, the World Meteorological Organization (WMO) notes that the number of deaths associated with heat will double in the next 20 years. During the period 2002–2010, Mexico recorded 393 deaths by excessive natural heat, mainly in the northeastern part of the country, where a significant positive association were found between temperature and heat stroke mortality [26,27].

Other diseases associated with CC are asthma that affected approximately one third of the child population [28], pneumonia, bronchopneumonia, and scorpion stings are among the first 20 causes of morbidity [29]. A study on the impact of climate variables on the incidence of scorpion stings was carried out by climate regions in central Mexico. It was found that the regions with the highest temperatures showed the greatest impact, with 9.8% increase (CI 95%: 8.3%–11.3%) for each 1°C increase in temperature [24]. These Mexican regions reported an average of 570 scorpion sting weekly cases and nearly 40% of them occurred in children under 18 years old [30].

## Adaptation Strategies in Mexico

Mexico has strong action on CC based on a General Law of Climate Change that establishes the basis for legal frameworks, financing, and the promotion of a low carbon economy. The Law created the Special Climate Change Program for the period 2014–2018. The executive body of the Program is the Mexican Inter-Ministerial Committee on Climate Change, which coordinates plans, programs, mitigation policies and strategies to implement national adaptation measures and health sector adaptation policies, such as the establishment of an early warning system, enhancing health sector capacities and increasing health promotion actions [31]. In addition, each state has CC Action Plans that include health risk issues based on regional diagnosis. However, the incorporation of health outcomes has been unequal and needs to be strengthened further.

## Final Remarks

Mexico is signatory of the Paris Agreement of the United Nations Framework Convention on Climate Change. However, CC scenarios in Mexico show significant increases in temperature, especially in the north, and changes in precipitation patterns in basically all regions [32]. The population that is currently in its childhood and their descendants will inherit these scenarios. Potential health impacts include greater heat stroke risks, food security issues, new vector niches, and combined effects of short-lived climate pollutants and atmospheric pollutants in large cities. Mexico’s researchers have documented present and future impacts, although studies are still insufficient. Mitigation and adaptation efforts must be intensified if we are to offer future generations better health conditions and well-being.
